# Highly Expressed FOXF1 Inhibit Non-Small-Cell Lung Cancer Growth via Inducing Tumor Suppressor and G1-Phase Cell-Cycle Arrest

**DOI:** 10.3390/ijms21093227

**Published:** 2020-05-02

**Authors:** Chia-Yu Wu, Chun-Hao Chan, Navneet Kumar Dubey, Hong-Jian Wei, Jui-Hua Lu, Chun-Chao Chang, Hsin-Chung Cheng, Keng-Liang Ou, Win-Ping Deng

**Affiliations:** 1 Division of Oral and Maxillofacial Surgery, Department of Dentistry, Taipei Medical University Hospital, Taipei 11031, Taiwan; borgiawu@gmail.com; 2 School of Dental Technology, College of Oral Medicine, Taipei Medical University, Taipei 11031, Taiwan; 3 School of Dentistry, College of Oral Medicine, Taipei Medical University, Taipei 11031, Taiwan; harry811003@gmail.com (C.-H.C.); bioengineer.nkd@gmail.com (N.K.D.); hjwei@tmu.edu.tw (H.-J.W.); rita890322@yahoo.com.tw (J.-H.L.); g4808@tmu.edu.tw (H.-C.C.); 4 Stem Cell Research Center, College of Oral Medicine, Taipei Medical University, Taipei 11031, Taiwan; 5 Division of Gastroenterology and Hepatology, Department of Internal Medicine, Taipei Medical University Hospital, Taipei 11031, Taiwan; chunchao@tmu.edu.tw; 6 Division of Gastroenterology and Hepatology, Department of Internal Medicine, School of Medicine, Taipei Medical University, Taipei 11031, Taiwan; 7 Department of Dentistry, Taipei Medical University Hospital, Taipei 11031, Taiwan; 8 Department of Dentistry, Taipei Medical University-Shuang Ho Hospital, New Taipei City 23561, Taiwan; klou@tmu.edu.tw; 9 3D Global Biotech Inc., New Taipei City 22175, Taiwan; 10 Graduate Institute of Basic Medicine, Fu Jen Catholic University, New Taipei City 24205, Taiwan

**Keywords:** FOXF1, lung cancer, cell migration, tumor suppressor, cell cycle

## Abstract

Cancer pathogenesis results from genetic alteration-induced high or low transcriptional programs, which become highly dependent on regulators of gene expression. However, their role in progressive regulation of non-small-cell lung cancer (NSCLC) and how these dependencies may offer opportunities for novel therapeutic options remain to be understood. Previously, we identified forkhead box F1 (FOXF1) as a reprogramming mediator which leads to stemnesss when mesenchymal stem cells fuse with lung cancer cells, and we now examine its effect on lung cancer through establishing lowly and highly expressing FOXF1 NSCLC engineered cell lines. Higher expression of FOXF1 was enabled in cell lines through lentiviral transduction, and their viability, proliferation, and anchorage-dependent growth was assessed. Flow cytometry and Western blot were used to analyze cellular percentage in cell-cycle phases and levels of cellular cyclins, respectively. In mice, tumorigenic behavior of FOXF1 was investigated. We found that FOXF1 was downregulated in lung cancer tissues and cancer cell lines. Cell proliferation and ability of migration, anchorage-independent growth, and transformation were inhibited in H441-FOXF1^H^ and H1299-FOXF1^H^, with upregulated tumor suppressor p21 and suppressed cellular cyclins, leading to cell-cycle arrest at the gap 1 (G1) phase. H441-FOXF1^H^ and H1299-FOXF1^H^ injected mice showed reduced tumor size. Conclusively, highly expressing FOXF1 inhibited NSCLC growth via activating tumor suppressor p21 and G1 cell-cycle arrest, thus offering a potentially novel therapeutic strategy for lung cancer.

## 1. Introduction

According to the National Cancer Institute, among all types of cancer, lung cancer is the second common cancer type with the highest mortality rate in the United States [1]. It is also considered the leading cause of death in Taiwan [2]. Of the two major types, non-small-cell lung cancer (NSCLC) is the most common, which is estimated to be 80% of lung cancer, whereas another major type is called small-cell lung cancer (SCLC), which is approximated to be 20%. Furthermore, the NSCLCs are sub-divided into adenocarcinoma (30–40%), squamous cell carcinoma (30–40%), and large-cell carcinoma (<10%) [3]. Notably, lung cancer has high risk of relapse after the surgery, as many cases fail to achieve a sufficient cure following surgery [4], and 30%–55% of patients with NSCLC die of the recurrence of lung cancer, despite curative resection [5,6]. Therefore, to eliminate lung cancer, an attempt was made to combine chemo- and radiotherapy, which might decrease the risk of relapse. However, some researches showed the inadequacy of this combined therapy [7,8,9]. Hence, developing a comprehensive treatment of lung cancer is still an important issue worldwide.

Genetic alterations induce high or low transcriptional programs, which lead to cancer pathogenesis. These programs may render cancer cells to become highly dependent on regulators of gene expression. Therefore, with reference to forkhead box F1 (FOXF1), a protein implicated in cancer progression, we aimed to understand outcomes of transcriptional dependencies and whether this protein involved in transcriptional control could be an attractive target of a new generation of drugs, thereby imparting opportunities for novel therapeutic interventions in cancer.

In recent years, FOXF1 proteins were implicated in cancer progression. These proteins are highly expressed in fetal and adult lung tissues [10], and they were identified to regulate angiogenesis during embryonic development [11]. Furthermore, FOXF1 also plays a vital role in the development of pulmonary alveoli and vasculature, whereas any deletions or mutations in the FOXF1 gene are believed to cause alveolar capillary dysplasia with misalignment of pulmonary veins [12,13,14]. In a few previous studies, FOXF1 was demonstrated as a tumor suppressor [15,16], and it was found downregulated in certain types of cancers, including prostate and breast cancer tissues, when compared to normal [17,18]. In our previous study, FOXF1 was identified as a reprogramming mediator that leads to stemness, when mesenchymal stem cells (MSCs) fuse with lung cancer cell, and its restoration leads to p21-regulated growth suppression in fusion progeny [19]. This implies that FOXF1 and its downstream molecules might act as molecular targets for the development of diagnostic and therapeutic tools against lung cancer. In this study, we initially examined FOXF1 expression in lung cancer tissues and cell lines compared to those with normal tissues. Furthermore, we created a highly expressing FOXF1 lung cancer cell line (FOXF1^H^) through lentiviral transduction of lowly expressed FOXF1 parental cell lines (FOXF1^L^) and comparatively characterized their cell proliferation and migration ability. We also attempted to determine mechanistic insight into FOXF1-mediated regulation of the cell cycle via flow cytometric analysis. Later, the transformation ability of these cell lines was tested in in vitro and in vivo models.

## 2. Results

### 2.1. FOXF1 is Downregulated in Lung Cancer Tissue and Cell Lines

**Figure 1:**
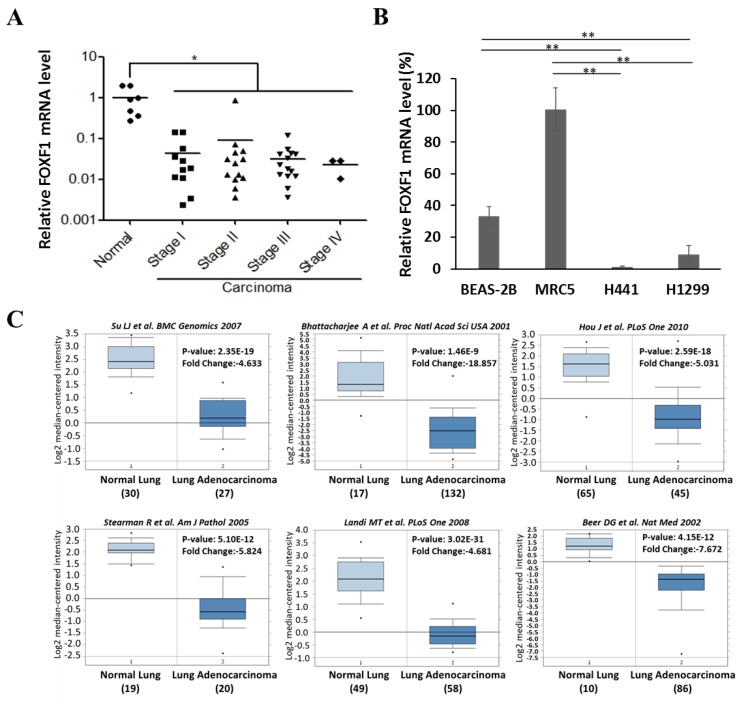
Downregulation of forkhead box F1 (FOXF1) in lung cancer. (**A**) Real-time PCR-dependent FOXF1 messenger RNA (mRNA) levels in lung cancer (*n* = 41) and normal lung tissues (*n* = 7). The relative mRNA level was stratified at dot plots according to the cancer grade. * *p* < 0.01, using Welch’s unpaired *t*-test. (**B**) FOXF1 mRNA levels in BEAS-2B, MRC5, H441, and H1299 cells. (**C**) In silico analysis of FOXF1 expression using Oncomine Research Edition (https://www.oncomine.org/). FOXF1 mRNA expressions in normal and malignant lung specimens are presented as box and whisker plots. The authors, published journal, and year of each study are indicated under the graph. The number of the sample (listed under each specimen), fold changes of the mRNA expression of FOXF1, and *p*-value are indicated in each panel. Data are expressed as means ± standard error (SE). * *p* < 0.05; ** *p* < 0.01, using Welch’s unpaired *t*-test.

Previously, our studies identified FOXF1 as a putative tumor suppressor, which could mediate mesenchymal stem cell fusion-induced reprogramming of lung cancer cells to a more benign state [19]. To verify the expression of FOXF1 in lung cancer compared to normal tissue, various stages of lung cancer tissue were collected. Additionally, FOXF1 expression in lung cancer cell lines (H441 and H1299) was compared with normal lung cell lines (MRC5 and BEAS-2B). Our results demonstrated significantly lowered gene expression of FOXF1 not only in cancer tissues (Figure 1A), but also in H441 and H1299 cell lines (Figure 1B), compared to their respective normal controls. As a result, highly proliferated cells with enlarged nucleus and nucleo-cytoplasmic ratio could be seen in lung histological sections of stage III and IV (Figure S1, Supplementary Materials). Furthermore, we also interrogated the FOXF1 expression in the Oncomine database (http://www.oncomine.org), a cancer microarray database and web-based data-mining platform, which provides publicly available gene expression datasets. We selected six studies [20,21,22,23,24,25] in this database which also revealed significantly lowered expression (4–19-fold) of FOXF1 gene in lung cancer compared to normal tissue (Figure 1C). A similar trend of FOXF1 expression profiles was also validated in The Cancer Genome Atlas (TCGA) and genotype-tissue expression (GTEx) projects using the GEPIA2 online platform (http://gepia2.cancer-pku.cn/#index) (Figure S2, Supplementary Materials).

### 2.2. Highly Expressed FOXF1 Lung Cancer Cell Showed Inhibited Cell Proliferation Ability

**Figure 2:**
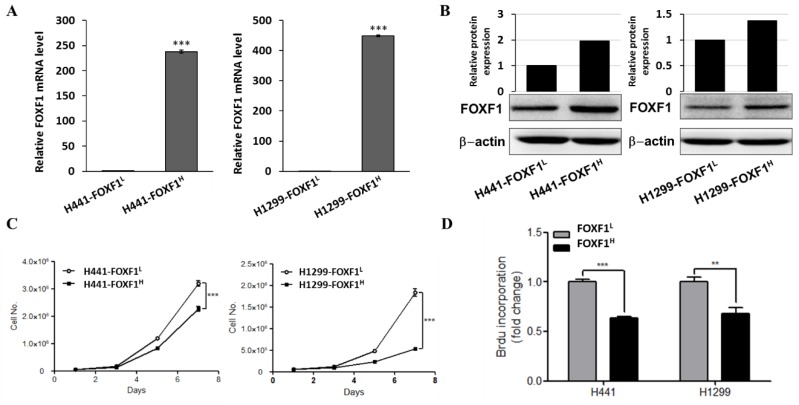
Proliferation ability of highly expressing FOXF1 non-small-cell lung cancer cell lines. Determination of relative expression levels of (**A**) FOXF1 mRNA, (**B**) protein, (**C**) cell growth, and (**D**) BrdU incorporation assay-based in vitro cell proliferation of H441-FOXF1^H^ and H1299-FOXF1^H^ cell lines compared to their respective controls (H441-FOXF1^L^ and H1299-FOXF1^L^). β-actin served as a loading control. Data are expressed as means ± SE. ** *p* < 0.01; *** *p* < 0.001, using Welch’s unpaired *t*-test.

After identifying the lowered expression of FOXF1 in lung cancer tissues and cell lines, we further established highly expressing FOXF1 lung cancer cell line (FOXF1^H^) through lentiviral transduction of lowly expressing FOXF1 parental cell lines (FOXF1^L^) to investigate the relationship between FOXF1 and lung cancer. Our qPCR and Western blot results confirmed the significantly higher expression of FOXF1 in these developed cell lines (Figure 2A,B, respectively). Thereafter, a cell counting and BrdU incorporation assay was conducted to assess the cell proliferation ability of H441-FOXF1^H^ and H1299-FOXF1^H^ cell lines. These cell lines showed reduced cell number (Figure 2C), with inhibited proliferation ability (Figure 2D), compared to their counterparts (H441-FOXF1^L^ and H1299-FOXF1^L^, respectively).

### 2.3. Highly Expressed FOXF1 Promotes G1 Cell-Cycle Arrest

**Figure 3:**
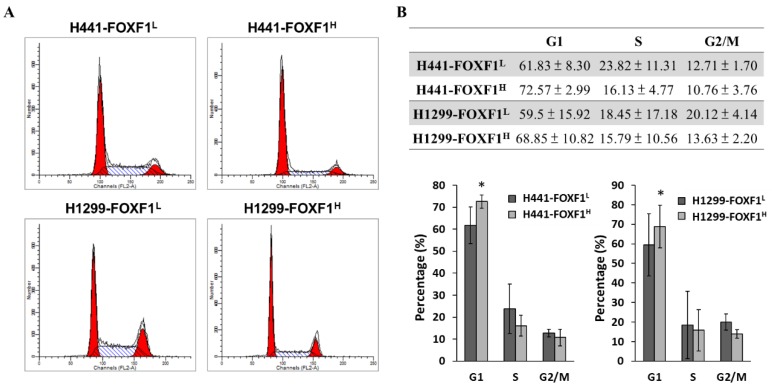
Effect of high expression of FOXF1 on cell cycle of non-small-cell lung cancer cell lines. (**A**) Representative histograms of flow cytometric analysis of cell cycle, and (**B**) quantification of cells (%) at the gap 1 (G1), synthesis (S), and gap 2 (G2)/mitosis (M) phase of H441-FOXF1^H^ and H1299-FOXF1^H^ cell lines compared to their respective controls. Data are expressed as means ± SE. * *p* < 0.05, using Welch’s unpaired *t-test*.

The cell-cycle checkpoints are signal transduction pathways to track the successful completion of events in a phase of the cell cycle [26]. Therefore, using flow cytometry, we further investigated the impact of FOXF1 expression on the cell cycle. The representative cell-cycle histograms showed the population shift to the G1 phase of H441-FOXF1^H^ and H1299-FOXF1^H^ cell lines (Figure 3A). The bar graph and the respective table are presented with the cellular percentage of each phases, which clearly show a higher population of cells in G1 phase of highly expressing FOXF1 cell lines compared to their counterparts (Figure 3B). This indicates that FOXF1 may promote pronounced cell-cycle arrest in the G1 phase of lung cancer.

### 2.4. High Expression of FOXF1 Promotes Tumor Suppression and Inhibits Cellular Cyclins

**Figure 4:**
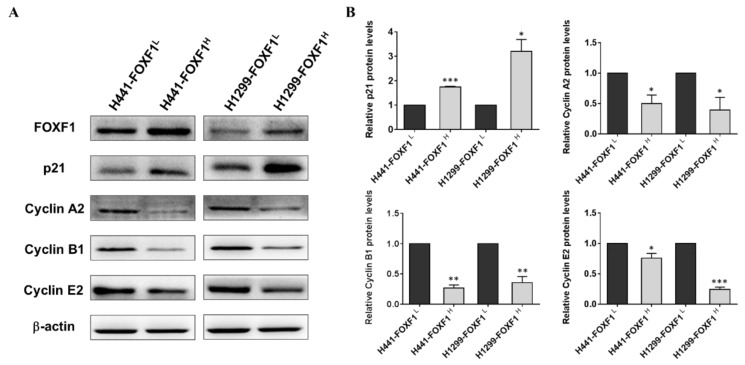
FOXF1-mediated regulation of cell-cycle regulatory proteins. (**A**) Representative Western blots showing expression of FOXF1, p21, and cyclins A2, B1, and E2 in highly FOXF1-expressing non-small-cell lung cancer (NSCLC) cell lines (H441-FOXF1^H^ and H1299-FOXF1^H^). (**B**) Quantified protein expressions of p21 and cyclins A2, B1, and E2. β-actin served as a loading control. Data are shown as means ± SE. *, *p* < 0.05; ** *p* < 0.01; *** *p* < 0.001, using paired *t*-test, compared to respective control.

The cell cycle is known to be regulated by tumor suppressors and cellular cyclins [27]. Hence, to gain mechanistic insight into FOXF1-mediated regulation of the cell cycle, we determined the protein expression of cell-cycle-related proteins, including p21, cyclin A2, cyclin B1, and cyclin E2. The Western blot demonstrated upregulated p21 level, a well-known promoter of cell-cycle arrest, in the H441-FOXF1^H^ and H1299-FOXF1^H^ cell lines. On the contrary, the levels of cyclin A2, B1, and E2 in these cell lines were inhibited (Figure 4A). The above-mentioned data were further confirmed through their quantification (Figure 4B).

### 2.5. High Expression of FOXF1 Inhibits Anchorage-Independent Growth Ability and Transformation Ability

**Figure 5:**
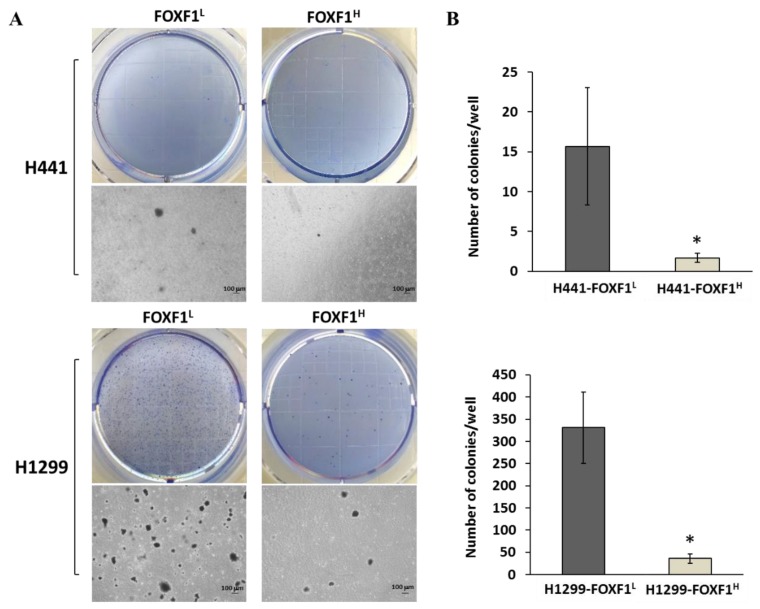
Analysis of anchorage-independent growth ability of highly expressing FOXF1 NSCLCs. Comparative phase-contrast images of (**A**) H441-FOXF1^H^ and H1299-FOXF1^H^ colonies with low-expressing counterparts and their relative quantifications (**B**). Images were captured at 10× magnification. Data are shown as means ± SE. * *p* < 0.05, using Welch’s unpaired *t*-test.

The ability of anchorage-independent cell growth and the ability of transformation are the identified signatures of tumors with metastatic potential [28]. Therefore, we also evaluated the other cancerous characteristics of highly expressing FOXF1 lung cancer cell lines, H441-FOXF1^H^ and H1299-FOXF1^H^. Specifically, we conducted the soft agar assay to observe the effect on ability of anchorage-independent growth and transformation of these cell lines, which showed significantly reduced colony number (Figure 5A,B), implying inhibited transformation ability.

### 2.6. High Expression of FOXF1 Inhibits Lung Cancer Cell Migration Ability

**Figure 6:**
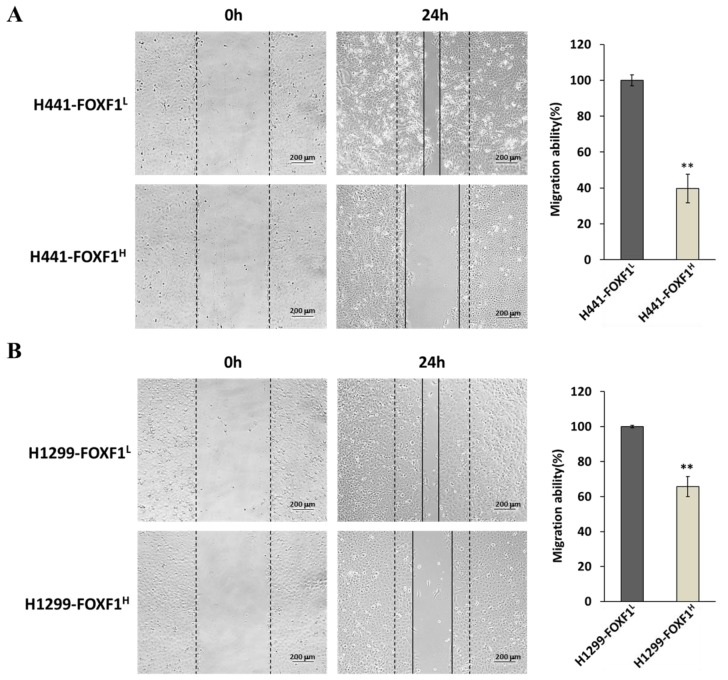
The effect of FOXF1 on the migration ability of non-small-cell lung cancer cell lines. The representative images and the migration area quantify the highly expressing FOXF1 cell lines and controls of H441 (**A**) and H1299 (**B**). Images were captured at 10× magnification. Data are shown as means ± SE. ** *p* < 0.01, using Welch’s unpaired *t*-test.

To evaluate the effect of high expression of FOXF1 on lung cancer cell metastatic potential, we performed the wound healing assay, which shows the migration ability of cell lines. Here, 24 h after wound creation, an inhibited migration ability was observed in H441-FOXF1^H^ (Figure 6A) and H1299-FOXF1^H^ (Figure 6B), which was also confirmed through quantification, compared to their respective controls.

### 2.7. High Expression of FOXF1 Inhibits Lung Cancer Cell Tumorigenicity In Vivo

**Figure 7:**
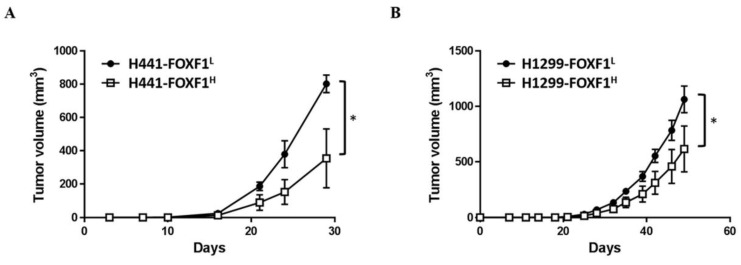
The in vivo anti-tumor effect of FOXF1 on non-small-cell lung cancer cell lines. Volumes of tumors generated by subcutaneous injection of highly expressing FOXF1 or control cell lines of (**A**) H441 and (**B**) H1299. Results are plotted as tumor volume versus days after implantation. * *p* < 0.05, using two-way ANOVA.

In order to determine the in vivo effect of FOXF1 on the tumorigenic ability of lung cancer cells, the highly expressing FOXF1 cells (H441-FOXF1^H^ and H1299-FOXF1^H^) were subcutaneously injected into non-obese diabetic/severe combined immunodeficiency (NOD-SCID) mice to observe the size of tumor formed. The results showed a significantly reduced tumor volume in the H441-FOXF1^H^ and H1299-FOXF1^H^ group compared to control (H441-FOXF1^L^ and H1299-FOXF1^L^) (Figure 7A,B), indicating that FOXF1 could inhibit tumorigenesis.

## 3. Discussion

FOXF1 is crucial to the development of the lung, and its haploinsufficiency may cause lung deformity [12,29], such as severe alveolar capillary dysplasia with misalignment of pulmonary veins [12,13,14]. FOXF1 is also reported as the downstream target of the hedgehog signaling pathway [30,31], which is a pivotal factor for cell differentiation and organ formation during embryogenesis. However, the hedgehog signaling pathway is aberrantly activated in various cancers, leading to cancer initiation, as well as tumor growth [32,33]. Being a downstream target of the hedgehog signaling pathway, many studies suggested that FOXF1 is positively correlated with cancer development. This is supported by a few reports, in which the expression of FOXF1 was increased in basal cell carcinoma, medulloblastoma, and rhabdomyosarcomas [34,35]. In a seminal study, FOXF1 was suggested as a potential prognostic marker due to its correlation with malignancy and metastasis of colorectal cancer [36]. A similar outcome was reported by Fulford et al., in which FOXF1 promoted prostate tumor growth and progression by activating extracellular signal-regulated kinase 5 (ERK5) signaling [37]. Even an immunohistochemical staining-based study demonstrated positively correlated FOXF1 expression in many NSCLCs with lymph node metastasis [38]. On the contrary, the functional role of FOXF1 remains controversial, as various studies also demonstrated that FOXF1 expression was inhibited in various tumor types including lung, prostate, bladder, ovarian, and breast cancers [15,17,18]. These pathogenic outcomes might be attributed to genetic alterations that induce high or low transcriptional programs, resulting in a dynamic network with multiprotein complexes collaborating as nodes of stimulating, suppressing, remodeling, and insulating function. In spite of this complexity, certain oncogenic impulses may depend on protein complexes, as well as individual factors; therefore, identifying and validating these targets could provide not only mechanistic insights, but also therapeutic options.

This above-mentioned evidence implies the different roles of FOXF1 in various types of cancers. Nonetheless, most of the clinical NSCLC samples demonstrated in our study exhibited a low expression of FOXF1, which was validated through the Oncomine database, as well as GEPIA2 online platform. Moreover, other studies also reported lowly expressed FOXF1 in clinical NSCLC samples [39,40]. These outcomes are also in line with immunohistochemical (IHC) staining-based studies on clinical lung and breast cancer [18,40]. Additionally, our previous study demonstrated that MSCs fuse spontaneously with lung cancer cells, thereby potentially reprogramming the cells to a slow-growing, non-tumorigenic, and stem-like state. According to Wei et al., this might be attributed to a complementation of genetic defects, including upregulation of FOXF1 and p21, as well as restoration of normal terminal differentiation pathways [19]. This study also showed that FOXF1, in addition to acting as a reprogramming stemness regulator, could serve as a putative tumor suppressor, leading to p21-regulated growth suppression in fused progeny. This implies the anti-lung cancer activities of FOXF1; however, the detailed underlying mechanism needs to be investigated. Hence, we aimed to investigate outcomes of transcriptional dependencies using the FOXF1 gene in lung cancer. The above-mentioned studies are in agreement with our results showing lowly expressed FOXF1 in cancer tissues, as well as in H441 and H1299 cell lines, in addition to data obtained from ONCOMINE database and in The Cancer Genome Atlas (TCGA) and genotype-tissue expression (GTEx) projects. However, no significant difference in relative FOXF1 expression was observed among lung cancer patients on the basis of gender, age, histopathological type, histologic grade, and tumor, node, metastasis (TNM) staging system in the groups of our tissue array data (Table S1, Supplementary Materials). It is well known that enhanced cell viability and accelerated proliferation are hallmarks of cancer. However, in this study, we reported an inhibited proliferation of highly expressing FOXF1 lung cancer cells compared to their relatively low-expression counterpart, which reveals the anti-proliferative activities induced by FOXF1. As per our previous study, the reprogrammed inhibition of FOXF1 in the fusion cell lines (MSCs with lung cancer cells) led to an inhibited p21 expression, which resulted in their accelerated grow rate [19]. Therefore, we infer that FOXF1 modulates lung cancer growth via regulating p21. It was documented that p21 participates in multiple tumor suppressor pathways and promotes anti-proliferative activities, which are independent of the classical p53 tumor-suppressor pathway [41]. Moreover, p21 is also reported as a universal inhibitor of cyclin kinases [42]. Cyclins are a family of proteins which control cell-cycle progression through activating cyclin-dependent kinase (CDK) enzymes [43]. According to the classical model of cell-cycle control, D-type cyclins and CDK4 or CDK6 regulate events in early G1 phase, whereas cyclin E–CDK2 triggers the synthesis (S) phase. Additionally, cyclin A–CDK1 and cyclin A-CDK2 regulate the completion of the S phase, while cyclin B-CDK1 is responsible for mitosis [44]. The arrest of the G1 phase of the cell cycle is an irreversible process, which is indicative of apoptotic cells [45]. These findings support our results, displaying upregulated levels of p21 and the inhibited cyclins A2, B1 and E2, leading to cell-cycle arrest at the G1 phase in the highly expressing FOXF1 cell lines. This is also in line with various studies demonstrating cell-cycle-associated regulation of cancer; consequently, cell-cycle inhibitors like FOXF1 might be considered as a therapeutic target in the management of cancer [46,47,48]. Furthermore, anchorage-independent growth is the capacity of transformed cells to grow independently of a solid surface, which is a hallmark of cancer [28,49]. In this context, our soft agar assay demonstrated a significantly inhibited anchorage-independent cell growth in highly expressing FOXF1 lung cancer cell lines, indicating the tumor-suppressing effect of FOXF1. Furthermore, to examine the in vivo effect of FOXF1 on tumor growth, we injected highly expressing FOXF1 cell lines H441-FOXF1^H^ and H1299-FOXF1^H^ in the mice, which revealed significantly decreased tumor size compared to their parental counterpart.

**Figure 8:**
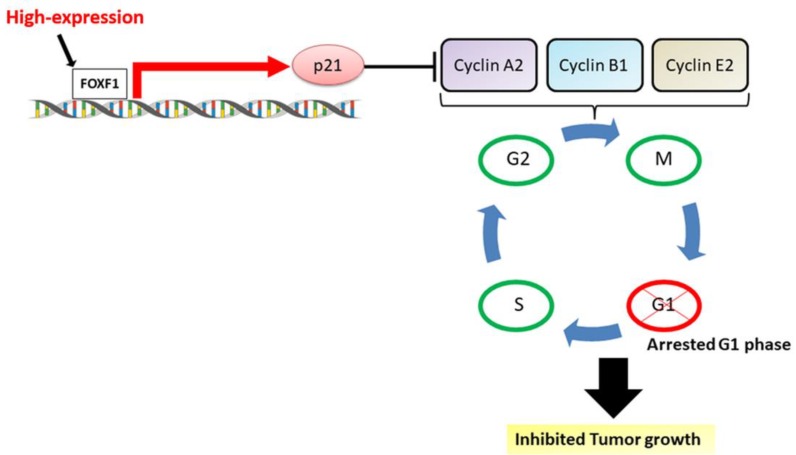
Schematic of possible mechanistic insight into FOXF1-inhibited non-small-cell lung cancer growth via inducing tumor suppressor and G1-phase cell-cycle arrest.

In addition to the various significant outcomes, this study also includes a few limitations. The observed downstream effects could be attributed to differential expression levels of FOXF1, as all the experiments were conducted under identical conditions using FOXF1^L^ and FOXF1^H^ cell lines; however, the role of any other possible confounding factor can be explored in future studies. Furthermore, although our study could not provide detailed insight into potential downstream targets of FOXF1, the recent ChIP-seq and RNA-seq analysis-based study by Bolte et al. revealed various FOXF1 targets, including the genes regulating extracellular matrix remodeling (Timp3, Adamts9) and cell-cycle progression (Cdkn1a, Cdkn2b, Cenpj, Tubb4a), which are crucial for lung regeneration [50]. This study also indicated that FOXF1 directly regulates Cdkn1a (p21) and Cdkn2b (p15) genes, which possess multiple FOXF1-binding sites near the promoter region and within introns. Moreover, overexpressed TIMP3 levels are found to be associated with inhibitory effects on cell invasion and migration in NSCLC [51]. Adamts9 was reported as a tumor suppressor which could inhibit tumor growth and angiogenesis in various cancers including lung cancer [15,52,53,54]. We further probed possible downstream targets of FOXF1 through TRRUST (Transcriptional Regulatory Relationships Unraveled by Sentence-Based Text Mining, www.grnpedia.org/trrust), a database of reference transcription factor (TF)–target regulatory interactions in humans based on literature curation [55]. This database includes an important study which reported E-cadherin (CDH1) as a downstream target of FOXF1. Specifically, FOXF1 could regulate the transcriptional activity of CDH1 by acting on its FOXF1-binding site, eventually contributing to cell migration and invasiveness in lung cancer [56]. Since cell polarity and normal epithelial structure are maintained by E-cadherin [57,58], its reduced levels would lead to increased cell motility and enhanced cancer cell invasion [59,60]. This evidence corresponds to our study where highly expressed FOXF1 could inhibit cell migration in lung cancer, possibly through upregulation of E-cadherin and TIMP3. Taken together, FOXF1 significantly inhibited the cell growth and migration of lung cancer, largely via stimulating tumor suppressor p21 and inhibiting levels of cyclins, leading to an arrested cell cycle in the G1 phase (Figure 8).

## 4. Materials and Methods

### 4.1. Cell Culture

H441 (ATCC^®^ HTB-174™) and H1299 (ATCC^®^ CRL-5803™) cell lines were cultured in Roswell Park Memorial Institute (RPMI)-1640 medium supplemented with 10% fetal bovine serum (GE healthcare life sciences, Logan, UT, USA) and 1% PSA (penicillin, streptomycin, and amphotericin) (Mediatech, Manassas, VA, USA). For highly expressing FOXF1 cell lines, H441-FOXF1^H^ and H1299-FOXF1^H^, an additional 2.5 μg/mL puromycin (Sigma-Aldrich, Saint Louis, MO, USA) was used in the medium. All the cell cultures were maintained at 37 °C in a humidified incubator with 5% CO_2_.

### 4.2. Animal Studies

All the animal studies were approved by The Institutional Animal Care and Use Committee (IUCAC) of Taipei Medical University (Approval no. LAC-2016-0526; 1 August 2017). Six-week-old non-obese diabetic/severe combined immunodeficiency (NOD/SCID) mice were purchased from the BioLAS Co., Taiwan. The mice were housed under pathogen-free conditions and fed autoclaved food and water. 

### 4.3. In Silico Analysis of FOXF1 Gene Expression

The Oncomine^TM^ Cancer Microarray Database (URL: www.oncomine.org, accession date: 10 December 2017) was used to perform the comparative in silico analysis of FOXF1 gene expression in cancer versus normal tissue [20,21,22,23,24,25]. Furthermore, we validated the FOXF1 expression profile in The Cancer Genome Atlas (TCGA) and genotype-tissue expression (GTEx) projects using the GEPIA2 online platform (URL: http://gepia2.cancer-pku.cn/#index, accession date: 10 April 2020). We chose datasets of lung adenocarcinoma (LUAD) and lung squamous cell carcinoma (LUSC), which were represented as box plots, with a *p*-value cutoff set at 0.05.

### 4.4. Higher Expression of FOXF1

Human FOXF1 open reading frame (ORF) complementary DNA (cDNA) was purchased (clone identifier (ID) #OHu23845) from GenScript and cloned into the pRNAT-U6.2-Lenti lentivirus expression vector from GenScript. Lentiviral vectors and packaging constructs were transfected into 293FT cells (Invitrogen) with Lipofectamine 2000 Transfection Reagent (Invitrogen). Infectious viral particles were collected 48 h after transfection. Log-phase target cells, including H441 and H1299 cells, were infected with appropriate virus titers in media containing 8 μg/mL polybrene. Media were changed the following day; 24 h later, infected cells were selected with 800 μg/mL of G418 (Invitrogen) for seven days and subsequently cultured in complete growth medium with 400 µg/mL G418. Real-time PCR and Western blot analyses were utilized to evaluate the level of FOXF1 expression.

### 4.5. Real-Time Polymerase Chain Reaction (PCR)

For lung cancer cell line analysis, total RNA was extracted using the PureLink RNA Mini Kit (Invitrogen, USA) according to the manufacturer’s instructions. Reverse transcription (RT) was performed as previously described [19]. Quantitative PCR was performed using an ABI 7300 real-time PCR system (Applied Biosystems), and gene expression was calculated using the 2^−ΔCt^ or 2^−ΔΔCt^ methods with calibration samples included in each experiment. For lung cancer tissue analysis, a TissueScan cDNA array plate (HLRT105) was purchased from Origene™ (Rockville, MD, USA), which consisted of seven-normal, six-stage IA, 5-IB, 13-IIB, 7-IIIA, 7-IIIB, and 3-IV. The sample patient population included 29 males and 19 females with age ranging from 44 to 84 years old.

The primers used were as follows:

β-actin-F (forward): 5′–AGAGCTACGAGCTGCCTGAC–3′;

β-actin-R (reverse): 5′–AGCACTGTGTTGGCGTACAG–3′;

FOXF1-F: 5′–AAGCCGCCCTATTCCTACATC–3′;

FOXF1-R: 5′–GCGCTTGGTGGGTGAACT–3′.

### 4.6. Western Blot Analysis

Protein extraction and immunoblotting were performed as previously described [19]. The following antibodies were used: rabbit polyclonal anti-FOXF1 (Abcam #ab23194, 1:500), rabbit monoclonal anti-p21 (Cell Signaling Technology #2947, 1:2000), mouse monoclonal anti-cyclin A2 (Cell Signaling Technology #4546, 1:1000), rabbit polyclonal anti-cyclin B1 (Cell Signaling Technology #4138, 1:1000), rabbit polyclonal anti-cyclin E2 (Cell Signaling Technology #4132, 1:750), and mouse monoclonal anti-β-actin (Millipore #MAB1501, 1:10000).

### 4.7. Cell Count

Control and highly expressing FOXF1 cell lines of H1299 and H441 were seeded at 5 × 10^4^ cells per well in a six-well plate and incubated for one, three, and five days. Cells were stained with 0.4% trypan blue (Invitrogen, Eugene, OR, USA) and counted with a hemocytometer after incubating for the indicated time course.

### 4.8. Cell Proliferation Assay

To measure the cell proliferation activity, both the lowly expressing FOXF1 control (H441-FOXF1^L^, H1299-FOXF1^L^) and the highly expressing FOXF1 lung cancer cells (H441-FOXF1^H^, H1299-FOXF1^H^) were seeded at a density of 1.6 × 10^4^ cells into 96-well plates. After 24 h of incubation, cell proliferation was determined in vitro using a BrdU cell proliferation assay kit (Merck Millipore Burlington, MA, USA). According to the manufacturer’s instructions, the optical density (OD) values at 450 nm wavelength were analyzed using a Multiskan PC (Thermo Labsystem, Beverly, MA, USA).

### 4.9. Cell-Cycle Analysis

For cell-cycle analysis by flow cytometry, the cells were trypsinized and washed with PBS and fixed with 75% ethanol. Then, 500 μL of RNase A (0.2 mg/mL, Sigma-Aldrich, 10109142001) and 500 μL of propidium iodide (0.02 mg/mL, Sigma-Aldrich, 11348639001) were added to the cell suspensions, and the mixtures were incubated for 30 min in the dark. A flow cytometer (BD FACS Calibur) was used for cell-cycle analysis, and 10,000 events for each sample were recorded. Data acquisition and analysis were done using BD FACSDiva software version 4.1 (BD Biosciences, San Jose, CA, USA), and the percentages of cells present in the G1, S, and G2/M (mitosis) phases were determined.

### 4.10. Anchorage-Independent Growth

Firstly, 1 mL of 0.5% agar in complete growth medium was added to each well of a six-well plate as a base agar. The top agar was prepared using 1 mL of 0.3% agar in complete growth medium containing 3 × 10^5^ cells of H441-FOXF1^L^ and H441-FOXF1^H^, and 1 × 10^4^ cells of H1299-FOXF1^L^ and H1299-FOXF1^H^, and it was overlaid on the base agar. Growth medium (2 mL) was added on top of the second layer and changed twice a week. After incubation for three weeks, the colonies formed were stained with 0.005% crystal violet in methanol (Fisher Scientific, Hampton, NH, USA) and then enumerated.

### 4.11. Wound Healing Assay

To evaluate the wound healing, 1.5 × 10^5^ cells of H441-FOXF1^L^ and H441-FOXF1^H^, and 1 × 10^5^ cells of H1299-FOXF1^L^ and H1299-FOXF1^H^ cells were seeded in a 24-well plate to obtain a confluent monolayer. The wounds were created through scraping the monolayer in a straight line with a 200-μL pipette tip, and debris was removed by washing with sterile PBS. Thereafter, the migration ability of these cells was assessed through measuring the recovered area of wound by cell migration.

### 4.12. Tumorigenicity In Vivo

To examine the tumorigenicity in vivo, 5 × 10^5^ cells of H441-FOXF1^L^ and H441-FOXF1^H^, and 1 × 10^6^ cells of H1299-FOXF1^L^ and H1299-FOXF1^H^ were subcutaneously injected into six-week-old non-obese diabetic/severe combined immunodeficiency (NOD/SCID) mice (*n* = 5). The tumor size was measured with a digital caliper twice a week. The tumor size was calculated using the following formula:

$$Volume=\frac{Lengh\times Width^{2}}{2}.$$

### 4.13. Statistical Analysis and Replicates

The sample size in each experiment was at least *n* = 3, unless otherwise indicated. Statistical analyses were conducted utilizing GraphPad Prism 5 (version 5.01, GraphPad Software, San Diego, CA, USA) and Microsoft Excel (Office 2016 Professional Plus, Santa Rosa, California, USA). All data are presented as means ± standard error (SE).

## 5. Conclusions

Based on our results, it could be inferred that highly expressing FOXF1 inhibits non-small-cell lung cancer growth via activating tumor suppressor p21, leading to cell-cycle arrest at the G1 phase. Thus, FOXF1 could be a potential therapeutic candidate for lung cancer.

## Supplementary Materials

Supplementary materials can be found at https://www.mdpi.com/1422-0067/21/9/3227/s1. Supplementary Table S1. Patient demographic and clinicopathological characteristics. Supplementary Figure S1. Representative lung tissue sections (H&E stain) of normallung and lung carcinoma (Stage III and IV). Supplementary Figure S2. *In silico* analysis of FOXF1 expression profiles from TheCancer Genome Atlas (TCGA) and genotype-tissue expression (GTEx) projects using GEPIA2 online platform.

## Abbreviations

FOXF1	Forkhead box F1
NSCLC	Non-small-cell lung cancer
