# Supplementary material for: Highly Expressed FOXF1 Inhibit Non-Small-Cell Lung Cancer Growth via Inducing Tumor Suppressor and G1-Phase Cell-Cycle Arrest
Source: Int J Mol Sci. 2020 May 2;21(9):3227. doi: 10.3390/ijms21093227 (PMC7246752; doi:10.3390/ijms21093227)
Supplement: Supplementary file 1 [file ijms-21-03227-s001.pdf]

Characteristics	No. of patients	Relative FOXF1 expression (Mean ± SEM)	P-value
**Gender**			0.1655
Male	24	0.070 ± 0.031	
Female	17	0.025 ± 0.005	
**Age (y)**			0.4031
≤65	20	0.036 ± 0.006	
>65	21	0.066 ± 0.035	
**Histopathology**			0.1457
Adenocarcinoma	22	0.058 ± 0.034	
Squamous cell carcinoma	13	0.053 ± 0.010	
Large cell carcinoma	6	0.024 ± 0.005	
**Histologic grade**			0.3753
Well differentiated (G1)	3	0.018 ± 0.006	
Moderately differentiated (G2)	13	0.034 ± 0.009	
Poorly differentiated (G3)	18	0.073 ± 0.041	
Unknown	7	0.044 ± 0.013	
**Primary Tumor (T-stage)**			0.8337
T1	9	0.043 ± 0.013	
T2	17	0.030 ± 0.005	
T3	7	0.144 ± 0.103	
T4	6	0.027 ± 0.007	
Tx	2	0.018 ± 0.001	
**Regional Lymph Nodes (N-stage)**			0.4063
N0	20	0.076 ± 0.037	
N1	10	0.024 ± 0.006	
N2	7	0.039 ± 0.009	
N3	1	0.019	
Nx	3	0.020 ± 0.003	
**Overall stage**			0.9648
I	11	0.038 ± 0.010	
II	13	0.089 ± 0.056	
III	14	0.031 ± 0.006	
IV	3	0.033 ± 0.008	

Supplementary Table 1. Patient demographic and clinicopathological characteristics.

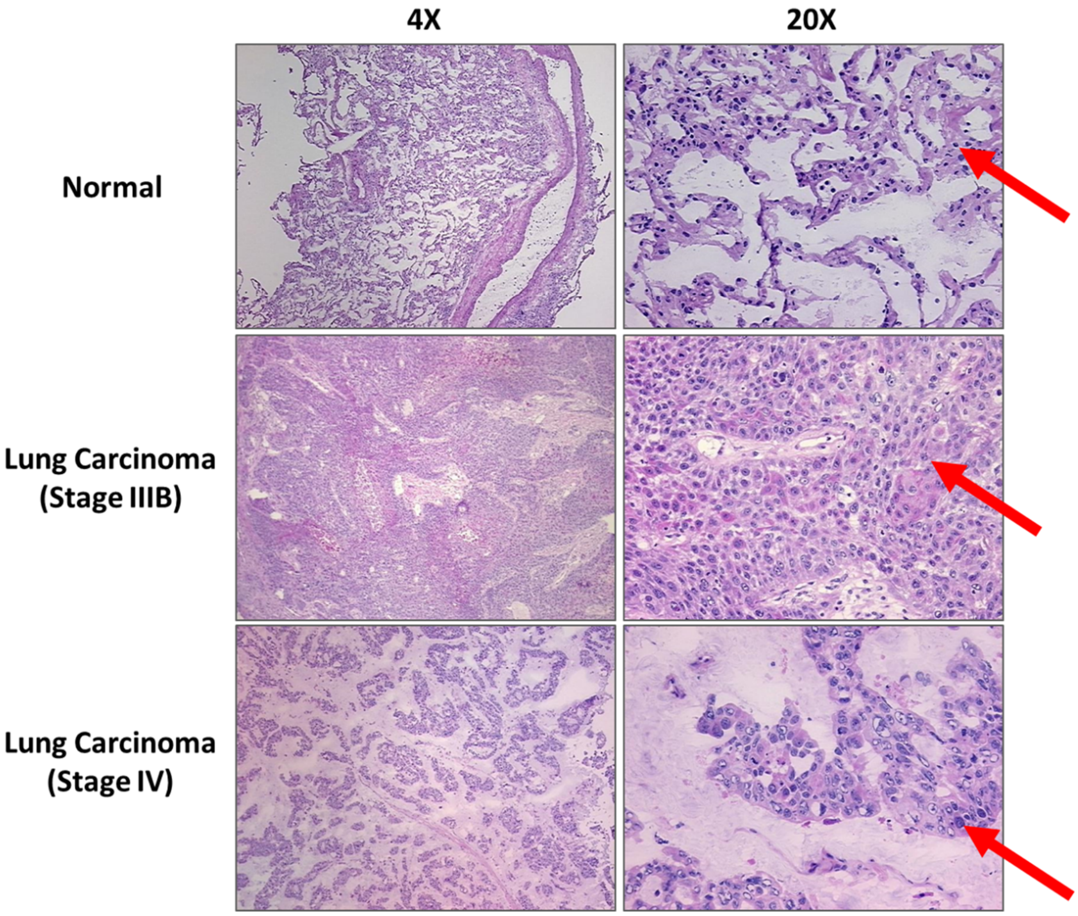

Supplementary Figure 1. Representative lung tissue sections (H&E stain) of normal lung and lung carcinoma (Stage III and IV). Images are captured at 4X and 20X magnification. The data are provided from Origene company (https://www.origene.com/catalog/tissues/tissuescan/hlrt105/tissuescan-lung-cancer-cdna-array-v)

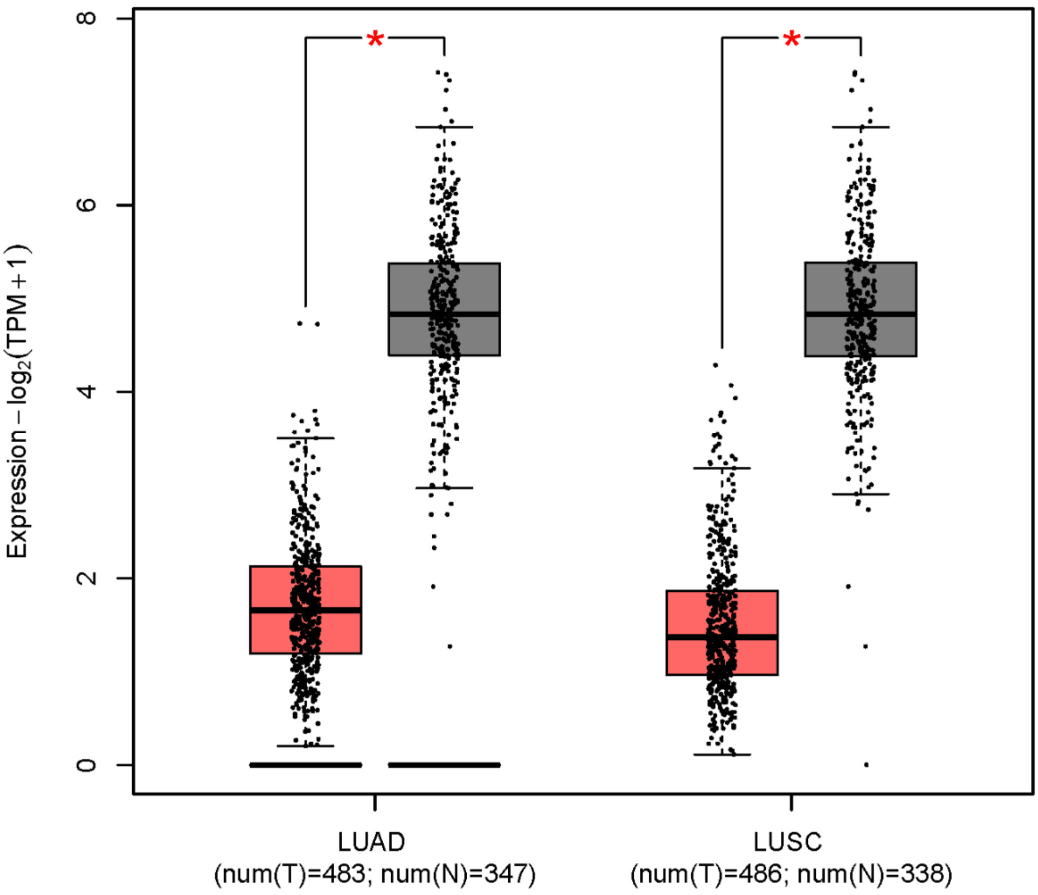

Supplementary Figure 2. *In silico* analysis of FOXF1 expression profiles from The Cancer Genome Atlas (TCGA) and genotype-tissue expression (GTEx) projects using GEPIA2 online platform (http://gepia2.cancer-pku.cn/#index). The FOXF1 expression of lung adenocarcinoma (LUAD) and lung squamous cell carcinoma (LUSC) are represented as box plot, and *p-value* cutoff is set at 0.05.
